# Cholemic Nephropathy as Cause of Acute and Chronic Kidney Disease. Update on an Under-Diagnosed Disease

**DOI:** 10.3390/life11111200

**Published:** 2021-11-06

**Authors:** Francesca Tinti, Ilaria Umbro, Mariadomenica D’Alessandro, Silvia Lai, Manuela Merli, Annalisa Noce, Nicola Di Daniele, Sandro Mazzaferro, Anna Paola Mitterhofer

**Affiliations:** 1 Nephrology Unit, Department of Translational and Precision Medicine, Sapienza University of Rome, 00185 Rome, Italy; ilaria.umbro@uniromail.it (I.U.); silvia.lai@uniroma1.it (S.L.); sandro.mazzaferro@uniroma1.it (S.M.); 2 Clinical Pathology Unit, Department of General Surgery “P.Stefanini”, Sapienza University of Rome, 00161 Rome, Italy; mariadomenica.dalessandro@uniroma1.it; 3 Gastroenterology Unit, Department of Translational and Precision Medicine, Sapienza University of Rome, 00185 Rome, Italy; manuela.merli@uniroma1.it; 4 UOC of Internal Medicine—Center of Hypertension and Nephrology Unit, Department of Systems Medicine, University of Rome Tor Vergata, Via Montpellier 1, 00133 Rome, Italy; annalisa.noce@uniroma2.it (A.N.); nicola.didaniele@uniroma2.it (N.D.D.); 5 Nephrology and Dialysis Unit, Department of Systems Medicine, University of Rome Tor Vergata, Via Montpellier 1, 00133 Rome, Italy

**Keywords:** cholemic nephropathy, acute kidney injury, chronic kidney disease, liver transplantation, acute on chronic liver failure, bile casts, promoting factors

## Abstract

Cholemic nephropathy (CN) is a recognized cause of acute kidney injury (AKI) in patients with severe hyperbilirubinemia (sHyb) and jaundice. Pathophysiological mechanisms of CN are not completely understood, but it seems caused both by direct toxicity of cholephiles and bile casts formation in nephrons enhanced by prolonged exposure to sHyb, particularly in the presence of promoting factors, as highlighted by a literature reviewed and by personal experience. The aim of our update is to retrace CN in its pathophysiology, risk factors, diagnosis and treatment, underlining the role of sHyb, promoting factors, and CN-AKI diagnostic criteria in the different clinical settings associated with this often-concealed disease. Our purpose is to focus on clinical manifestation of CN, exploring the possible transition to CKD. Cholemic nephropathy is an overlooked clinical entity that enters differential diagnosis with other causes of AKI. Early diagnosis and treatment are essential because renal injury could be fully reversible as rapidly as bilirubin levels are reduced. In conclusion, our proposal is to introduce an alert for considering CN in diagnostic and prognostic scores that include bilirubin and/or creatinine with acute renal involvement, with the aim of early diagnosis and treatment of sHyb to reduce the burden on renal outcome.

## 1. Introduction

Cholemic nephropathy (CN) is a recognized cause of acute kidney injury (AKI) in patients with severe hyperbilirubinemia (sHyb) and jaundice.

The association between renal dysfunction and sHyb was first described in 1899 by Quincke and Nothnagel in patients with jaundice, kidney injury and evidence of bile casts in their kidney biopsies [1]. However, the causal link between jaundice and kidney injury was suggested in 1922 by Haessler et al. who observed urine sediment of humans and dogs with jaundice [2].

Since then, the term “cholemic nephrosis” was introduced but, over the years, it has been replaced by several synonyms such as “bile or biliary nephrosis”, “jaundice-related nephropathy”, “bile acid nephropathy”, and the most recent term “bile cast nephropathy” [3].

Cholemic nephropathy is a little-known and overlooked diagnosis. In literature it is generally described in patients with hyperbilirubinemia caused by obstructive jaundice, malignancy, drug-induced liver injury and acute hepatitis, more rarely in patients with advanced liver diseases such as decompensated cirrhosis or end stage liver disease [3].

Acute kidney injury developing in cholemic nephropathy is secondary to sHyb; its early recognition may lead to improved diagnosis, management and enhanced outcome of renal function.

## 2. Cholemic Nephropathy—Pathophysiology

Pathophysiological mechanisms of CN are not completely understood. It seems to be caused both by direct toxicity of cholephiles and bile casts formation in nephrons, with epithelial cell damage and tubular obstruction, the typical feature of bile casts CN, associated to interstitial nephritis [4].

Under physiological conditions, the kidney takes part in the regulation of the bile acid pool size. Around 10–50% of the reabsorbed bile acids present in bile escape hepatic extraction, spill over to the peripheral circulation, and approximately 100 μm of non-protein bound bile acids per day are subjected to glomerular filtration under physiological conditions [5]. Filtered bile acids are nearly completely reabsorbed in the proximal tubule by a sodium-dependent mechanism via the apical sodium-dependent bile acid transporter, then the effective amount of urinary excreted bile acids ranges around 1–2 μmol per day [5,6,7,8].

**Figure 1:**
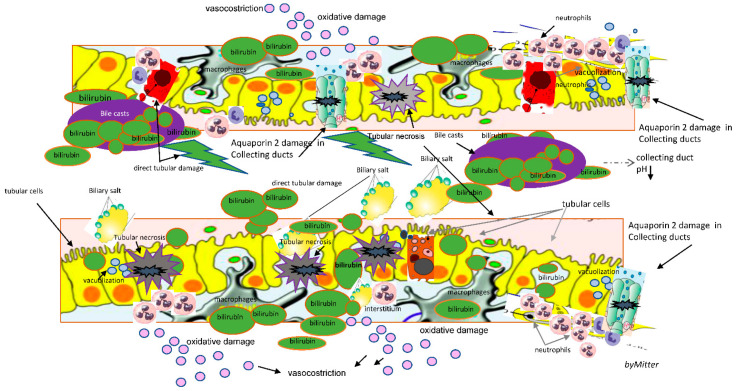
Schematic description of features characterizing cholemic nephropathy: renal tubular, interstitial and epithelial damage with bile casts formation, direct tubular toxicity damage, bile interstitial nephropathy, bile casts formation and direct kidney injury, oxidative stress, direct renal epithelial cells apoptosis and necrosis, reduced production of nitric oxide; tubular toxicity with tubular cells necrosis and exfoliation, interstitial nephritis, impaired M2 macrophages most involved in wound healing and fibrosis.

The most recent published evidence from animal studies highlights a pivotal role for bile acids and biliary constituents in CN, that could have a role in this specific type of renal injury [9,10] Figure 1.

It is not clear whether bilirubin has a direct toxic effect on the kidney; however, bilirubin levels correlate well with levels of bile acids [11].

Enhanced urinary excretion of bile acids in cholestasis results from excess amount of filtered bile acids beyond the maximum capacity for tubular reabsorption and enhanced tubular secretion [12,13]. This may cause renal injury. As such, bile acids were hypothesized to directly cause oxidative damage to tubular cell membranes with subsequent release of vasoactive mediators that in turn affect renal function through renal vasoconstriction and thus reducing glomerular filtration rate (GFR) [14,15].

In an autopsy study that examined histologic changes in 75 patients in the setting of liver necrosis various kidney lesions were demonstrated. These included the following: (1) glomerular congestion, (2) proximal and distal tubule vacuolization and necrosis, and (3) interstitial edema and cellular infiltration [16]. In these cases, an unexpected presence of profound biliary pigment and casts in the kidneys was found [16]. Besides tubular epithelial injury, basement membrane defects leading to leaky tubules and obstruction of collecting ducts due to sloughed cells and bile casts were found, with damage to Aqaporin-2 channels [17] Figure 1.

On experimental kidney biopsies in *common bile duct ligated (CBDL) mice*, a model of tubular epithelial injury of cholestasis, CN was observed predominantly on day 3 on collecting ducts. Despite difficult evaluation of collecting ducts, the role of tubular acidosis that increases along the tubule at this level, mostly seems involved in bile casts formation [12].

Although pathophysiological mechanisms of bile casts formation are not fully understood, renal dysfunction in obstructive jaundice has been attributed to the tubular effect of bile and to hemodynamic changes induced by portal and systemic endotoxemia [18,19]. In contrast, histopathological studies on kidneys from patients who died of renal failure during jaundice have shown morphological alterations of tubular cells, suggestive of direct renal damage [20].

Prolonged exposure to severe hyperbilirubinemia defined by total bilirubin higher than 20 mg/dL (sHyb), enhances bile casts formation and direct kidney injury, especially in presence of bile casts promoting factors (BCPFs) such as metabolic acidosis and hypoalbuminemia, often overlooked in this setting [21,22]. Metabolic acidosis, clinically defined by a serum bicarbonate concentration <22 mmol/L [23] and hypoalbuminemia are typical features of chronic kidney disease (CKD) and liver failure (LF), respectively. These seem to be critical parameters involved in the regulation of renal tubular bile acid transport in the early phase of obstructive cholestasis and, in conditions of sHyb, seem to promote the intratubular formation of bile casts, tubular damage and impaired renal function development [12,21,22].

Hypoalbuminemia may contribute to the development of hypovolemia and reduction in the renal blood flow, enhancing tubular bilirubin and biliary casts formation [21]. Low serum albumin concentration may also increase the amount of free bilirubin (bilirubin fraction not linked to albumin) that can cross glomerular basement membrane and pass into the urine. Acidosis may enhance the intratubular formation of bile casts consisting of desquamated epithelial cells, precipitated proteins and bilirubin, in particular at the level of collecting duct, where the reduced solubility of bile acids is favoured by acidosis [20]. Moreover, in specific systemic settings associated with sHyb other factors may contribute to bile casts formation and kidney damage.

In a study on renal biopsies performed in cirrhotic patients, all patients with CN were positive for bilirubin in the urine and similarly urobilinogen, a degradation product of bilirubin [24].

The potential nephrotoxic effect of bilirubin has been attributed to the bilirubin accumulation in mitochondria with subsequent inhibition of oxidative phosphorylation with decreased adenosine triphosphatase activity. This is associated with mitochondrial defects and increased permeability of cell membranes, resulting in modified electrolyte content and cell volume [25,26,27].

Otherwise, the specific role of bilirubin in CN remains controversial [28]. In experimental models, bilirubin has been shown to act rather renoprotectively as an antioxidant with effects on improvement of vascular resistance, tubular dysfunction, mitochondrial integrity and inhibition of nicotinamide adenine dinucleotide phosphate hydrogen (NADPH) oxidase and nitric oxide synthase 2 (NOS2) expression [28,29,30,31,32].

In liver diseases, further different elements should be considered in promoting bile casts development in addition to hypoalbuminemia. In a liver transplant setting, immunosuppressive antirejection therapy such as calcineurin inhibitors drugs (CNI) are responsible for renal tubular acidosis with normal anion gap, due to the reduction of bicarbonate reabsorption with no evidence of parenchymatous renal disease [33]. It has been demonstrated that the absorption of bicarbonate by cortical collecting ducts in rats treated with CNI is reduced to 30% compared to normal [34].

Recently, numerous clinical and experimental studies have shown a prominent role of oxidative stress in CNI nephrotoxicity. It has been demonstrated in several cellular models that cyclosporine (CsA) enhances mitochondrial reactive oxygen species (ROS) synthesis in tubular cells, inducing modifications in mitochondrial physiology and decreases levels of antioxidant systems such as glutathione, vitamin C and E. In addition, mitochondria have a role in numerous cellular functions: intracellular calcium control, gluconeogenesis, fatty acid synthesis and cellular apoptosis. As a result, mitochondrial ROS can induce direct renal epithelial cells apoptosis [35].

It is recognized that CNI have distal tubular effects, in addition to the effects caused by the reduced production of nitric oxide [34,36,37].

Furthermore, CsA is known to upregulate TGF-β production in renal proximal tubular epithelial cells and this may contribute to tubular damage [36]. An experimental study on human immortalized proximal tubular epithelial cells demonstrated that CsA, azathioprine and methylprednisolone have a less protective effect compared to MMF and rapamycin on epithelial to mesenchymal transition leading to formation of myofibroblasts, leading to a greater susceptibility to damage such as bilirubin or bile acids [33].

## 3. Cholemic Nephropathy—Diagnosis, Acute Kidney Injury (AKI) and Chronic Kidney Disease (CKD)

**Figure 2:**
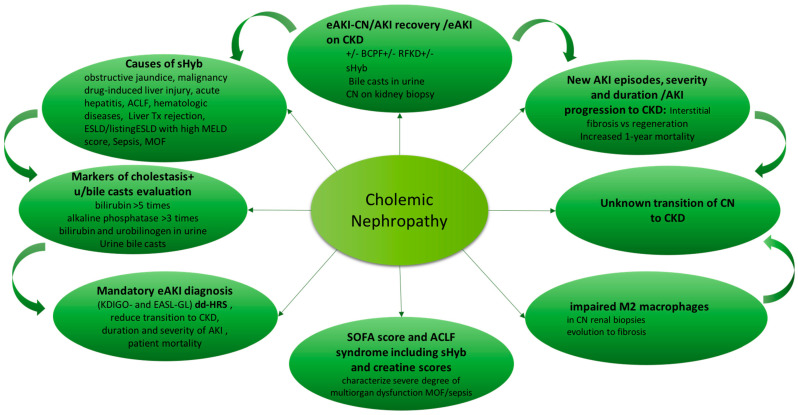
Cholemic nephropathy, AKI and CKD: Diagnosis of AKI related to CN and potential evolution of CN to CKD. Abbreviations: ACLF: Acute-on-Chronic Liver Failure; AKI: acute kidney injury; BCPF: bile cast promoting factors; CKD: chronic kidney disease; CN: cholemic nephropathy; dd-HRS: differential diagnosis with hepato-renal syndrome; eAKI: early AKI diagnosis; EASL: European Association for the Study of the Liver; ESLD: end-stage liver disease; listing ESLD: patients on the waiting list for liver transplantation; GL: guidelines; KDIGO: kidney disease: improving global outcome; M2 macrophages: macrophages most involved in wound healing; MELD score: Model for End Stage Liver disease score; MOF: multiorgan failure; sHyb: severe hyperbilirubinemia (>20 mg/dL); SOFA: sequential organ failure assessment.

Cholemic nephropathy diagnosis is based on sudden reduction of kidney function of normal or previous reduced kidney function (AKI or AKI on CKD) associated with sHyb, presence of bile casts in urine and features of biliary nephropathy at kidney biopsy [20]. A diagram of diagnosis is reported in Figure 2.

An important percentage of patients who experience AKI, defined on KDIGO 2012 diagnostic criteria [38], do not return to normal renal function regardless of the cause of renal injury, especially when persisting risk factors of kidney damage or nephrotoxic causes [39]. In patients who recover renal function, the recovery process after AKI may be associated with interstitial fibrosis rather than regeneration, predisposing these patients to CKD [40].

The duration and severity of AKI predict progression to CKD and contribute to the unabated increase in the number of patients with CKD and end stage renal disease [41]. Acute Kidney Injury is associated with a higher risk of death compared to patients without AKI and this increased risk rises incrementally with the severity of AKI [40]. Patients with the diagnosis of AKI or AKI overlapping CKD have an increased 1-year mortality as compared with patients with CKD (*p* < 0.001) [24].

Although AKI is the typical feature of kidney dysfunction associated with CN, to the best of our knowledge, the potential evolution of CN to CKD has not clearly explored.

In literature, CN is frequently described in patients with sHyb caused by obstructive jaundice [3], malignancy [21], drug-induced liver injury and acute hepatitis [3].

In a cohort of 818 patients with cirrhosis, CN was observed on kidney biopsy in 18% of patients with AKI and repeated and prolonged AKI episodes correlated with CKD transition, but no correlation between CN and CKD was described [24].

In these patients, early AKI diagnosis on the basis of the recent Consensus EASL 2018 guidelines [42] is required, in particular for those cases with initial AKI development. In fact, early resolution of cholestasis responsible of sHyb, may lead to AKI recovery with reduction in frequency of urinary bile casts formation until total disappearance [16,19,43].

Markers of cholestasis, including bilirubin higher than five times normal levels, alkaline phosphatase higher than three times and as well as bilirubin and urobilinogen in urine are considered independent risk factors associated to CN diagnosis [17].

Prevalence of CN, characterized by tubular toxicity with tubular cells necrosis and exfoliation, bile cast formation and interstitial nephritis, has been evaluated in 29% out of 105 kidney biopsies performed in cirrhotic patients with AKI. In this large, prospective cohort study evaluating the impact of AKI on transition to CKD, ATN compared to other causes of kidney damage showed a higher risk of CKD [24].

Among risk factors evaluated, development of new AKI episodes, duration and severity of AKI were recognized as predicting factors for CKD transition. The high number of impaired M2 macrophages most involved in wound healing described in CN renal biopsies highlights the role in CN and AKI patients with elevated bilirubin, leading to severe fibrosis as already described in mice with CN [9].

Therefore, early diagnosis and treatment of AKI is mandatory, as suggested by KIDIGO guidelines [38]. Kidney dysfunction associated with sHyb should also be recognized in patients with sepsis and multiorgan involvement in septic shock, as defined by the sequential organ failure assessment (SOFA) score, where the severe cases of dysfunction with a score greater than 2 are associated with the higher bilirubin levels [44]. Similarly, kidney dysfunction is associated with sHyb in a distinct syndrome that develops in cirrhotic patients with acute decompensation of cirrhosis and jaundice, that is the acute-on-chronic liver failure (ACLF) [45]. Acute-on-chronic liver failure is a recently recognized syndrome characterized by liver decompensation associated to organ/system failure involving liver, kidney, brain, coagulation, circulation and/or respiration, associated with poor survival (28-day mortality rate 30–40%) [45]. Acute-on-chronic liver failure patients are characterized by acute onset of clinical signs in conjunction with sHyb and prothrombin time greater than 1.5 times the upper reference range, with or without evidence of hepatic encephalopathy and renal dysfunction [46]. Diagnostic criteria for ACLF includes the SOFA score in the evaluation, and recognize the severe cases of dysfunction (classification of score 4) for those patients with hyperbilirubinemia >12 mg/dL and creatinine with a range score 1–4 for serum creatinine (sCr) >1.2 mg/dL (CLIF-SOFA score). [45].

Mechanisms responsible for kidney damage in this setting are multiple and complex, including CN, and renal failure contributes significantly in these patients to the high mortality. It is conceivable that CN should be considered among the other causes of AKI included in the ACLF diagnosis. In particular, the evaluation of bile casts in urine should be encouraged in all cases of AKI, requiring or not RRT, and sHyb (score 4 for liver dysfunction) for early detection of an additional cause of kidney damage [47]. Recognition and treatment of CN are crucial in this setting, and are finalized to bilirubin reduction, symptoms withdrawal for prognosis improvement implications [48].

The ACLF score (CLIF C-ADs) was proved to be more accurate for predicting outcomes in acutely decompensated cirrhotic patients than other scores, such as the MELD or MELD-Na mortality score based on serum bilirubin, creatinine and INR and used preferably for listing and prioritization to liver transplant [24,47,49]. According to the CLIF C-ADs or MELD score, a correct evaluation of renal function is instrumental. Renal failure is common in patients with sepsis or a high ACLF or MELD score. In this regard, it is important to consider that renal biopsy is not easily performed in patients with liver disease due to impaired coagulation, but clinical diagnosis needs to be considered for a better patient’s outcome [24]. Therefore, clinical and laboratory criteria become extremely important and required for CN diagnosis in the context of specific settings not including kidney biopsy owing to coagulopathy with increased risk of bleeding [17].

In patients with CN the diagnosis of AKI is challenging especially in presence of advanced liver disease [20]. The spectrum of causes for AKI in end-stage liver disease (ESLD) or in acute decompensated liver disease includes many conditions such as hypovolemia due to gastrointestinal bleeding, aggressive diuretic treatment, diarrhea, hypotensive state, infections or fever, that are frequently associated with AKI. The differential diagnosis with hepatorenal syndrome (HRS) is also mandatory. In this setting, sCr laboratory dosage is linked to different biases such as reduced hepatic creatine synthesis, reduced skeletal muscle mass, and protein malnutrition. It is well known that in patients with liver dysfunction, mild degrees of renal impairment may remain undiagnosed. In patients with liver disease, standard creatinine-based formulas such as the MDRD have been shown to substantially overestimate true GFR by 30–40% (20–40 mL/min/1.72 m^2^), especially those with low GFR [45,50,51,52]. Moreover, the simultaneous presence of sHyb influences the laboratory method used. The high blood levels of bilirubin, as in case of CN, can spectrally but also chemically interfere in numerous reactions as an underestimation of sCr values in the Jaffè colorimetric reaction [50]. Although the enzymatic creatinine assay is reported to have less interference in a case of sHyb, a negative interference by bilirubin can be still observed [51]. This interference seems to be attributed to the consumption of peroxide in the initial reaction mixture [52].

## 4. Cholemic Nephropathy (CN)—Treatment 

Clinical management of CN treatment is based on bilirubin level reduction <20 mg/dL with two fundamental approaches:

1. Limit as soon as possible factors involved with bilirubin or bile acid tubular precipitation and toxicity (bile cast promoting factors);
2. Reduce bilirubin level and or bile acids in plasma and urine by treating the underlying cause of sHyb.

1. In order to decrease total bilirubin levels and limit its nephrotoxicity, vascular expansion with isotonic solutions or saline solutions is suggested. Unfortunately, results of efficacy are still lacking and hemodilution is not always feasible in patients with AKI. Intravenous albumin administration is considered useful to increase renal blood flow by enhancing hematic albumin levels when low, and to reduce the amount of free bilirubin that crosses glomerular basement membrane and pass into the urine. Metabolic acidosis should also be treated with bicarbonate administration in order to maintain a normal pH and reduce the intratubular formation of bile casts, in particular at collecting duct level where bile acids reduce solubility due to the acidic environment.

A pharmacological approach has been proposed with norursodeoxycholic acid (norUDCA) medication that seems to improve CN in bile duct-ligated mice [10].

In severe cases, efficacy of extracorporeal albumin dialysis (ECAD), including molecular adsorbent recirculating system (*MARS*) or single-pass albumin dialysis (*SPAD*) systems, has been proposed.

The two methods MARS and SPAD techniques, proved their feasibility and efficacy [53,54,55] to reduce bilirubin levels in plasma [56]. MARS and SPAD have been reported in patients with CN, acute-on-chronic or acute liver failure and/or with pruritus or intoxication [57,58,59]. If required, these two techniques can be coupled to conventional haemodialysis or continuous renal replacement therapies. Another technique available to perform bilirubin detoxification is the fractioned plasma separation, adsorption and haemodialysis (Prometheus). This system consists of two circuits: one for conventional high-flux haemodialysis, and one module for protein-bound substances depuration [60]. MARS, unlike SPAD, may also affect other biological parameters, such as creatinine and urea. [56]; the clearance of the creatinine is, however, moderate (−24 mmol/L/MARS session for a dialysate flow of 2 L/h).

The effects of ECAD on bilirubin levels are transitory and not a resolution. The aim of ECAD is exclusively to reduce endogenous albumin-bound toxins. Moreover, given the high cost of extracorporeal albumin-dependent methods, these are not considered as routine therapeutic options.

2. In the setting of CN diagnosis AKI is a complication of sHyb, therefore identifying the cause of sHyb is mandatory and propedeutic for specific therapy or a procedure to reduce sHyb and sometime to prevent kidney damage. In fact, as shown in experimental mouse model of CN, kidney function recovered with resolution of sHyb and disappearance of bilirubinuria [9,11].

In patients with chronic underlying liver disease and unavailability of specific drug therapy, CN did not resolve, as hyperbilirubinemia could not improve by medical intervention. Moreover, CN was only one of many disease-defining factors in those patients, and consequently, all progressed to end-stage renal disease requiring RRT [17].

The scenario linking AKI with sHyb is wide and several clinical conditions should be considered in the diagnostic path.

In obstructive jaundice, diagnosis and resolution of obstructive cause should be achieved as soon as possible to reduce bilirubin levels <20 mg/dL.

In other settings, such as sepsis, liver or hematological diseases, CN diagnosis is more complex and differential diagnosis have to consider diverse causes of jaundice.

In cirrhotic patients impairment of renal function often occurs. Severity of liver disease, as assessed by high MELD score (3 months mortality score), predicts higher risk of CKD, maybe related to the high values of creatinine and bilirubin, components of the score. In these patients, hepatorenal syndrome (HRS) is recognized as the most frequent cause of AKI [61] but among no-HRS causes, CN has been reported in case reports or autopsy studies. In contrast to previous evidence, CN was a frequent finding in patients with liver disease, AKI and sHyb as shown in a study on 79 patients with liver disease who were submitted to renal biopsy. Interestingly in these patients with sHyb, CN was commonly observed in kidney biopsies with loss of Aqaporin-2 expression in the collecting duct, maybe as a result of the toxic effect of cholestasis. All patients with CN were positive for bilirubin in the urine and presented a significant higher MELD score [17]. In all cases who recovered kidney function particular different diagnosis were performed and specific treatment to reduce available sHyb levels were delivered (i.e., lamivudine for hepatitis B, resolution of cholestasis in obstructive jaundice, steroids for autoimmune hepatitis, rifampicin for intrahepatic cholestasis, etc.) [17].

### Clinical Management of CN Treatment

Lack of specific therapeutic options remains an important limitation for clinical management. 

Resolving the cause of hyperbilirubinemia should be the primary aim of treatment, as removing the primary cause that triggers hyperbilirubinemia is the only effective causative treatment.

This should be feasible in case of anatomical obstruction of bile ducts, and more specific in cases of parenchymal liver disease, i.e., cholestatic liver diseases, liver cirrhosis or conditions of liver dysfunction in the context of systemic disease such as infections, sepsis and hematological disorders, rejection of liver transplantation, etc.

On the other side, early recognition and treatment of AKI is mandatory to avoid severe deterioration of renal function and needing of renal replacement therapy, leading to non-recovery of renal function. The study of biomarkers of early AKI damage, such as urine neutrophil gelatinase-associated lipocalin and interleukin-1β, is also proposed [62].

The course of diagnosis and treatment should follow several steps to recognize and manage different disorders occurring simultaneously.

The first and essential step is to identify the condition of hyperbilirubinemia and sHyb before this could exert the damage at renal level. Treatment of sHyb in the early phase before renal complication development should be considered to avoid renal involvement and to prevent AKI development.

Monitoring of renal function and evaluation of bile cast in the urine sediment are the second and essential steps to early recognize the involvement of kidneys before the occurrence of severe damage.

Contextually, identification and treatment of promoting factors such as hypoalbuminemia and metabolic acidosis should be achieved and considered as further step, as well as identification and treatment of concurrent causes of renal dysfunction often occurring, i.e., infections, dehydration and additional causes or renal hypoperfusions.

Recognition of AKI in the early phase, in particular of AKI stage 1, may leads to prompt management in acceptable conditions of the patient, before the occurrence of severe worsening to graver stages and needing of invasive treatments and dialysis.

Based on this diagnostic and therapeutic path, extracorporeal elimination of bilirubin by ECAD should be considered only in those cases of persistent intractable sHyb, as a bridge to resolution [63].

In fact, we can speculate that ECAD in the early phase of sHyb may prevent deposition of bile pigments in renal tubules avoiding the formation of bile casts. However, excessive costs and potential side effects represent strong limitations.

Excessive cost and logistic necessities are the primary limitation in using these techniques as standard treatment. Moreover, the necessities of placing an adequate vascular access to process the sufficient amount of blood may expose the patient to several side effects, i.e., bleeding or infections, in particular in those conditions linked to ESLD characterized by derangement of coagulation and low platelet count. Principal side effects linked to ECAD also regard the occurrence of decline of platelets levels and activation of coagulation [55].

Finally, ECAD effects on bilirubin levels are temporary and do not resolve this, limiting its application to candidates for treatment to resolve this issue, such as liver transplantation in ESLD patients or surgical correction of obstructive biliary disease.

The availability of vascular access may couple the removal of sHyb and treatment of severe AKI with conventional hemodialysis or continuous renal replacement therapy [60].

The early diagnosis of AKI in the context of sHyb associated to the detection of bile casts in urine sediment may also simplify the diagnosis of CN, leading to early treatment and avoiding the requirement of performing renal biopsy, procedure that may be risky or contraindicated in this kind of compromised patient, reserving the procedure to highly selected cases [62].

## 5. Cholemic Nephropathy and Revision of Literature

Most papers of CN consist of case reports, some revisions and few longitudinal studies. The diagnosis of AKI is mainly reported on the basis of sCr increase from baseline value or a decrease of urinary output, but not always classified according to KDIGO or EASL guidelines [38,42].

### 5.1. CN and AKI Diagnostic Criteria

The causes of sHyb most frequently reported result from drug-induced cholestasis, liver cirrhosis, obstructive jaundice and malignant cholangiocarcinoma [64], alcoholic hepatitis, severely cholestatic liver graft, organ failure secondary to infections, sepsis, all conditions that could be associated with CN [17,21,22,24,46,48,63,64,65,66]. The majority of cases reported serum bilirubin levels above 20 mg/dL with peaks of 58 mg/dL in one of the first case described in literature, highlighting the severity of hyperbilirubinemia [67].

The cases of AKI described in the context of sHyb usually correspond to the most severe cases, associated to the higher values of sCr reported or to the need for dialysis [3,16,48,63,64,65,66,67,68,69,70,71,72,73,74].

Few cases performed a renal biopsy and the diagnosis of CN resulted as an unexpected diagnosis, always in the context of hyperbilirubinemia. Sens et al. [63] described the case of a 37-year-old male with chronic liver disease secondary to a mutation in the transcription factor 2 hospitalized for sHyb and AKI. After exclusion of alternative causes of AKI, renal biopsy was performed showing CN; Rafat et al. [64] reported the case of a 46-year-old kidney transplant recipient admitted for progressive jaundice (total bilirubin >30.0 mg/dL) due to a malignant cholangiocarcinoma and deterioration of graft function. In this case, after exclusion of common pre- and post-renal causes of AKI, a kidney biopsy was performed and resulted diagnostic for CN. Patel et al. [48] reported the case of a 54-year-old man with sHyb (total bilirubin > 19 mg/dL) due to drug induced acute liver failure who developed oligoanuric kidney injury and underwent HD. Urine analysis was performed and revealed 3+ red blood cells, 5–10 white blood cells, positive leukocyte esterase and presence of eosinophils which suggested an immune-mediated etiology. Unfortunately, bilirubinuria and bile cast were not tested in the urine. The renal biopsy, however, was diagnostic for CN. Legris T et al. [65] have described a patient with *Leptospira interrogans* serovar with sHyb and AKI. Urinalysis was not performed nor a kidney biopsy. In this case, CN was not considered, and AKI was diagnosed as secondary to sepsis.

Further cases describe patients with oligoanuric AKI in the context of sHyb secondary to drug-induced cholestasis and infections, interpreting the occurrence of AKI as caused by sepsis but no bile casts on urine sediment was investigated and CN nephropathy was not hypothesized [65].

### 5.2. Bile Casts Promoting Factors (BCPFs) and Bile Casts Formation in CN

Bile cast promoting factors such as hypoalbuminemia or acidosis were reported in few cases, even in those patients with a histological diagnosis of CN. Levels of serum albumin were reported in very few papers [48,69,73,74], while arterial blood gas was shown in only three papers [64,66,71].

Three cases described AKI in the context of sHyb after liver transplantation [68,72,73]. In all cases the authors did not search for the presence of bile casts on urine examination neither evaluated the presence of BCPFs including CNI therapy. In two cases the authors considered other diagnostic hypotheses of AKI (HRS, sepsis) out of CN despite sHyb and the renal biopsy not being performed [68,73]. In the third case the renal biopsy was performed and the diagnosis of CN carried out on the basis of abundant pigmented casts positive on Hall’s stain noted in the distal tubules and loss of the brush borders in the proximal tubules [72].

One pediatric case was reported in the context of ACLF, with a patient who presented with ACLF and AKI secondary to CN with fatal outcome, diagnosed on autoptic kidney biopsy [71].

Interestingly, Luciano et al. [66] describes CN in a bodybuilder abusing anabolic androgenic steroids. Pigmented granular cast and bile-stained granular casts were detected at urine sediment; bile-stained granular casts were shown on a kidney biopsy within the distal tubules, associated with ultrastructural signs of cell injury with dilated mitochondrial cristae and bile acid accumulation in lysosome. Fisler et al. [70] described the case of a 56-year-old male with liver failure secondary to intramuscular anabolic steroids overdose with progressive AKI. Given the persistence of anuria, which required HD and the presence of bile casts on urine sediment, a kidney biopsy was performed showing CN.

### 5.3. CN and Microscopic Urine Analysis

The evaluation of urinary sediment was rarely reported, describing the presence of bile casts. Overall, urinalysis in those patients in which diagnosis of CN was made with renal biopsy is not known about. The CN diagnosis requires appropriate level of clinical suspicion to avoid overlooking and its consequently underreporting. Microscopic analysis of urine is a suitable non-invasive test representing the best surrogate for kidney histology [35].

Furthermore, urine microscopy is advantageous because of extensive availability, procedure simplicity and low costs. Sequeira et al. [69] described the case of a 41-year-old woman with alcoholic hepatitis, jaundice sHyb and oligoanuric AKI. Given the presence of bile casts in urine sediment they performed a renal biopsy diagnostic for CN. Pitlick et al. [74] reported two cases: one of a 37-year-old man with alcoholic hepatitis with sHy and oligoanuric AKI, and a second case of an 87-year-old man with drug-induced liver injury secondary to antibiotic use with a decline of renal function; urinalysis in both cases showed the presence of urobilinogen and bilirubinuria with granular casts and renal tubule epithelial cells on microscopic examination; renal biopsy confirmed CN. In another case report of AKI with jaundice described by Aniort et al. [16], microscopic examination of urine sediment was not performed and nor were blood gas analysis or albumin evaluation reported, but a kidney biopsy showed CN with several green casts in tubular lumens.

A more recent histopathologic study by Van Slambrouck et al. [3] surveyed kidney involvement, particularly bile cast formation, in 44 jaundiced patients. The liver injuries included advanced cirrhosis, cholestatic/obstructive jaundice, primary hepatic jaundice, and hemolytic jaundice. Bile casts were present in 61% of patients with cirrhotic jaundice and, interestingly, 100% of patients with alcohol induced cirrhosis. The presence of bile casts appeared associated with higher sCr concentrations and was associated significantly with higher total and direct bilirubin levels. Histologic evaluation showed that the presence of bile casts was more pronounced in distal nephron segments, but also noted within proximal tubules, especially in those with more sHyb. Acute tubular injury, which was present in 73% of patients, was characterized by attenuated tubular epithelial cytoplasm and loss of a proximal tubule brush border [3]. Authors concluded that CN is a common pathologic finding for kidney injury associated with severe liver dysfunction. In a recent cohort of patients with cirrhosis (818 patients) CN was observed in a kidney biopsy and on an autoptic study, in 18% of cases with AKI. In this cohort, repeated and prolonged AKI episodes correlated with CKD transition [17].

On the basis of literature, we would suggest an early diagnosis through clinical and biochemical criteria including microscopic analysis of urine searching for the presence of bile casts and, when possible, with renal biopsy.

## 6. Personal Experience

Here we describe a case of CN in a patient with acute liver failure after liver transplantation. Informed consent for publication of the data was obtained.

Case report. We describe the case of an 8-year-old Italian female child who underwent liver transplantation for biliary atresia in 1990, receiving immunosuppressive therapy with cyclosporine, azathioprine and steroids. At the age of 33 she voluntarily suspended immunosuppressive therapy and intense pruritus associated with jaundice appeared. On admission, skin and sclera were frankly jaundiced, with normal level of consciousness, transaminases 24 times above the upper limits of normality, GGT twice, ALP 4 times, total bilirubin of 4.38 mg/dL reaching progressively the peak level of 30.1 mg/dL associated with reduced liver protein synthesis activity with serum albumin (sAlb) of 2.8 g/L and INR 1.7, as for acute decompensation of the liver [45].

The hepatic and biliary ultrasound was normal. Her immunosuppressive therapy was immediately restored, and a first high dose steroid pulse (methylprednisolone 1 g) was administered followed by repeated high dose steroid bolus and oral prednisone on the basis of the liver biopsy performed, which confirmed the presence of severe acute rejection due to liver cirrhosis. Renal function, initially normal (sCr 0.8 mg/dL) and glomerular filtration rate (eGFR) estimated by MDRD (of 87.8 mL/min/1.73 m^2^ as basal value), showed rapid reduction to an eGFR of 39.5 mL/min/1.73 m^2^ (moving from G2 to G3b-eGFR category) with increase of sCr to 1.6 mg/dL in 48 h (2 times baseline), diagnostic for AKI stage 2 onset (KDIGO, EASL [38,42]) and a MELD-Na score of 24 [47]. 

**Figure 3:**
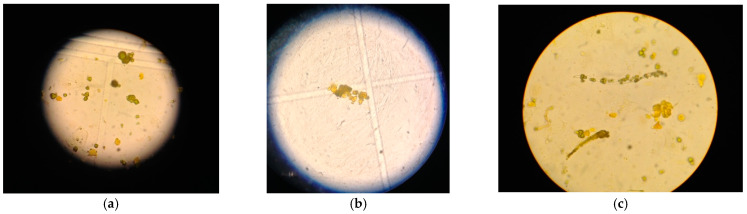
Bilirubin pigmented casts in urine (phase contrast microscope original magnification 400×). (**a**) bilirubin pigmented precipitates. (**b**) biliary cast containing renal epithelial cells. (**c**) biliary cast, pigmented renal epithelial cells, pigmented tadpole cell. For manual microscopic examination: 10 mL aliquot of the first urine of the morning was centrifuged at 400× *g* for 10 min, then the supernatant urine was removed, the sediment resuspended, transferred to a slide, and analyzed with a phase contrast microscope.

Urine analysis, normal on admission, showed bilirubinuria (6 mg/dL), bilirubin crystals, numerous bilirubin casts and white blood cells (10/µL) at microscopic examination of urine sediment as in Figure 3; the arterial blood gas test showed the presence of metabolic acidosis with normal anion gap: pH 7.34, pO_2_ 119 mmHg, pCO_2_ 30 mmHg, O_2_ Sat 98.4%, HCO_3_ 16.2 mmol/L, base excess −7.8 mmol/L, serum sodium 133 mmol/L, serum chloride 104 mmol/L. A kidney ultrasound showed altered renal medullary cortical differentiation related to increased cortical renal echogenicity, and a dynamic renal scintigraphy with 99 mTcDTPA revealed a bilateral reduction of the functional activity of the kidneys with decreased and slowed excretion of the radioisotope showing total measured GFR of 48 mL/min. The renal biopsy was not performed because of the worsening of liver protido-synthetic activity with impaired blood coagulation (INR 1.7), and clinical diagnosis of CN in a patient with renal-organ failure in the setting of ACLF was undertaken, on the basis of the presence of the following criteria: AKI with sHyb, bilirubin casts at urine microscopic examination associated with BCPFs (hypoalbuminemia, metabolic acidosis and CNI therapy in this case). We treated metabolic acidosis (i.v. sodium bicarbonate to correct bicarbonate deficiency 70 mEq), hydro-electrolyte balance and the hypoalbuminemia (continuous infusions of 20% human albumin, until correction) as well as graft rejection by increasing corticosteroid therapy. Her clinical conditions rapidly improved, an arterial blood-gas analysis test returned to normal pH and gas exchange, as well as serum albumin 30 g/L, showing simultaneous recovery of renal function with eGFR of 60.2 mL/min/1.73 m^2^ (returned to G2-GFR category 89–60 mL/min), sCr to1.11 mg/dL, and disappearance of bilirubin casts from urine.

At 6 months, ultrasound showed the kidneys had returned to normal cortico-medullary differentiation, serum albumin of 43 g/L, serum total bilirubin of 1.29 mg/dL, conjugated bilirubin of 0.99 mg/dL, INR of 0.89, sCr of 1.0 mg/dL with a sustained (>3 months) eGFR of 60.8 mL/min/1.73 m^2^ and negative urinalysis. 

Immunosuppressive therapy was optimized, introducing everolimus added to tacrolimus in order to reduce nephrotoxicity and preserve renal function.

**Figure 4:**
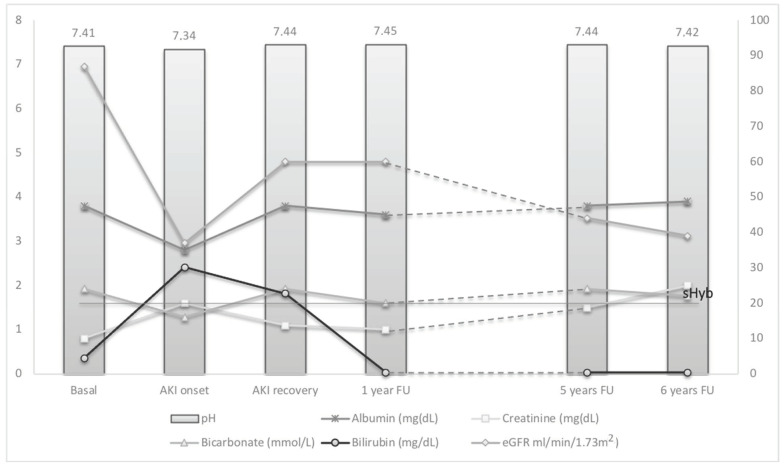
Trend of liver and renal function associated to the description of bile cast promoting factors.

Despite apparent recovery from AKI sustained for 36 months, sCr showed a progressive increasing, reaching the value of 2.0 mg/dL after 5 years. Figure 4.

In our patient, we found the presence of metabolic acidosis with low serum pH and [HCO_3_-] (7.34 and 16.2 mmol/L, respectively) with a normal anion gap, suggesting the possible presence of tubular acidosis.

Despite recovery of renal filtration after AKI, the follow up demonstrated a slight but progressive deterioration of renal function over 5 years, with decreasing measured GFR at 39 mL/min/1.73 m^2^, despite good control of conditions associated with renal damage progression.

In conclusion, in our patient, recovery of renal function with early CN diagnosis, treatment of BCPFs and early specific treatment of causes of sHyb has been instrumental, before further worsening of kidney function.

The AKI episode was associated with severe hyperbilirubinemia (>20 mg/dL), hypoalbuminemia and metabolic acidosis, during treatment with cyclosporin A. The resolution of hyperbilirubinemia and the correction of promoting factors restored the renal function with the disappearance of urinary sediment alterations (bilirubinuria and bilirubin casts). Despite recovery of renal filtration after AKI, the follow up demonstrated a slight but progressive deterioration of renal function over 5 years, with decreasing measured GFR at 39 mL/min/1.73 m^2^, despite good control of conditions associated with renal damage progression.

## 7. Discussion

Given the low number of patients with AKI and sHyb who underwent kidney biopsy both in liver setting or in patients with comorbidities, CN is likely underdiagnosed [24]. 

Early identification and treatment of CN are crucial in order to obtain resolution of symptoms and improvement of prognosis. An important percentage of patients who experienced AKI do not return to normal renal function, underlying the role of AKI on the development of CKD, regardless of the cause of the injury.

CN is a little-known and overlooked clinical entity that enters differential diagnosis with other causes of AKI in particular in liver disease. Early diagnosis and treatment are essential because in this setting renal injury could be fully reversible as rapidly as bilirubin levels are reduced. The importance of diagnosis on the basis of clinical criteria, including research of bile casts in urine and bilirubinuria, should be underlined.

Prolonged exposure to high levels of total bilirubin, usually higher than 20 mg/dL, enhances bile casts formation and direct renal tubular injury, especially in the presence of low serum albumin concentration, as in our case. Hypoalbuminemia have been recognized as BCPF, that is a favoring condition in CN [2].

High levels of bilirubin (cut off >20 mg/dL) are considered crucial in indicating the evolution from the physiologic to the pathologic renal response at which tubular injury occurs.

Most of the data reported in literature on patients with CN were derived from case reports or autopsy studies.

Kidney impairment is multifactorial, and diagnosis is strongly conditioned by the clinically variable context.

Despite the fact that in obstructive deeply jaundiced patients the acute onset of kidney dysfunction can be easily diagnosed and recognized as CN, in other settings CN diagnosis is more difficult and unfocused.

The European Association for the Study of the Liver (EASL) published a clinical practice guideline on the management of patients with decompensated cirrhosis, including definitions of kidney disease [42]. AKI is a common complication in patients with liver cirrhosis, and HRS as a cause of AKI (HRS-AKI) occurs in approximately 20% of hospitalized patients with decompensated liver cirrhosis [75].

Other causes of AKI (non-HRS-AKI) include prerenal causes including bleeding, diuretic treatment but also inflammation, bacterial translocation, cardiac dysfunction in advanced cirrhosis, hypotension, hypoperfusion etc. In this context, CN should be more considered. In fact, in this setting despite the frequent presence of hyperbilirubinemia, CN diagnosis remains a submerged disease.

In conclusion our proposal is to introduce an alert for considering CN in diagnostic and prognostic scores that include bilirubin and/or creatinine with acute renal involvement. This suggestion should direct clinicians to consider an early CN diagnosis and treat high bilirubin levels as soon as possible with specific treatment, including albumin dialysis system to obtain a possible renal recovery.

Cirrhotic pathophysiology undoubtedly contributes to kidney injury, but a high load of filtered bile acids also seems to induce tubular injury, perhaps in cells that have been primed by kidney ischemia. Bile acids are reabsorbed into tubular cells (primarily proximal) and likely impair cell function and structure. This is evidenced by the presence of exfoliated tubular cells (swollen and vacuolated) seen on urine microscopy and tubular cells with distorted mitochondria on kidney biopsy [66].

Pathophysiological mechanisms of CN are not completely understood, AKI seems to be related to acute tubular injury caused by sHyb and bile/acid bile casts damage on nephrons [3].

The great amount of bilirubin filtered by glomeruli exceeds the maximum capacity of reabsorption by tubules and enhances tubular secretion due to structural changes of tubular epithelium and mitochondria leading to modified electrolyte content and cell volume, pH changes and oxidative stress resulting in bile cast formation and deposition, tubular toxicity and injury [3,12].

Indeed, experimental and human data support the idea that bile acids have a direct role in causing AKI [3].

The main contribution of our work is that we highlighted the underdiagnosis of CN that can be a reversible condition, and the need for an early diagnosis and specific treatment. CN is considered a cause of AKI but transition to CKD can be considered, especially in the persistence of kidney damage.

During an episode of acute graft rejection, our 33-year-old liver transplant recipient developed a CN in the absence of apparent pre-existing renal dysfunction. This case shows that acute liver dysfunction-induced sHyb in a liver transplant recipient is associated with formation of bile casts and occurrence of AKI, in the context of liver transplantation. 

We also highlighted the importance of searching for urine bile cast in our AKI case associated with jaundice, and in revised cases of the literature, focusing on the presence of bile casts, BCPF and their early treatment. With our results and from the evaluation of the literature we have been able to underline the importance of a systematic approach in all cases of AKI with jaundice to early diagnose features of CN, which is a frequent condition, often overlooked.

Close cooperation among hepatologists, nephrologists and nephropathologists may help to diagnose CN correctly.
